# Outcomes and modifiable resuscitative characteristics amongst pan-Asian out-of-hospital cardiac arrest occurring at night

**DOI:** 10.1097/MD.0000000000014611

**Published:** 2019-03-08

**Authors:** Andrew Fu Wah Ho, Ying Hao, Pin Pin Pek, Nur Shahidah, Susan Yap, Yih Yng Ng, Kwanhathai Darin Wong, Eui Jung Lee, Pairoj Khruekarnchana, Win Wah, Nan Liu, Hideharu Tanaka, Sang Do Shin, Matthew Huei-Ming Ma, Marcus Eng Hock Ong

**Affiliations:** a SingHealth Emergency Medicine Residency Programme, Singapore Health Services, Singapore; b Division of Medicine, Singapore General Hospital; c Department of Emergency Medicine, Singapore General Hospital, Singapore; d Medical Department, Singapore Civil Defence Force, Singapore; e Emergency Department, Hospital Pulau Pinang, Georgetown, Pulau Pinang, Malaysia; f Department of Emergency Medicine, Seoul National University Hospital, Seoul, Korea; g Department of Emergency Medicine, Rajavithi Hospital, Bangkok, Thailand; h Unit for Prehospital Emergency Care, Singapore General Hospital, Singapore; i Health Services Research Centre, Singapore Health Services, Singapore; j Centre for Quantitative Medicine, Duke-NUS Medical School, Singapore; k Department of Emergency System, Graduate School of Sport System, Kokushikan University, Tokyo, Japan; l Department of Emergency Medicine, National Taiwan University Hospital, National Taiwan University, Taipei, Taiwan; m Health Services & Systems Research, Duke-NUS Medical School, Singapore.

**Keywords:** automated external defibrillator, bystander cardiopulmonary resuscitation, circadian patterns, out-of-hospital cardiac arrest

## Abstract

Studies are divided on the effect of day-night temporal differences on clinical outcomes in out-of-hospital cardiac arrest (OHCA). This study aimed to elucidate any differences in OHCA survival between day and night occurrence, and the factors associated with differences in survival.

This was a prospective, observational study of OHCA cases across multinational Pan-Asian sites. Cases were divided according to time call received by dispatch centers into day (0700H–1900H) and night (1900H–0659H). Primary outcome was 30-day survival. Secondary outcomes were prehospital and hospital modifiable resuscitative characteristics.

About 22,501 out of 55,881 cases occurred at night. Night cases were less likely to be witnessed (40.2% vs. 43.1%, *P* < .001), more likely to occur at home (32.5% vs. 29%, *P* < .001), had non-shockable initial rhythms (90.8% vs. 89.4%, *P* < .001), lower bystander CPR rates (36.2 vs. 37.6%, *P* = .001), lower bystander AED application rate (0.3% vs. 0.7%, *P* < .001), lower rates of prehospital defibrillation (13% vs. 14.4%, *P* < .001), and were less likely to receive prehospital adrenaline (9.8% vs. 11%, *P* < .001). 30-day survival at night was lower with an adjusted odds ratio of 0.79 (95% CI 0.73–0.86, *P* < .001). On multivariate logistic regression, occurrence at night was associated with decreased provision of bystander CPR, bystander AED application, and prehospital adrenaline.

30-day survival was worse in OHCA occurring at night. There were circadian patterns in incidence. Bystander CPR and bystander AED application were significantly lower at night in multivariate analysis. This would at least partially explain the decreased survival at night.

## 1 Introduction

Out-of-hospital cardiac arrest (OHCA) is a global health concern.^[1]^ In the Asia-Pacific, the incidence of OHCA has been rising, due to the advent of lifestyle diseases and an increasingly aging population. OHCA survival rates in the Asia-Pacific are generally low, ranging from 2% to 11%.^[2]^ Favorable outcomes in OHCA hinges on the rapid commencement and seamless provision of a set of rescuer actions, at the levels of the bystander, emergency medical services (EMS), and hospital emergency care providers.^[3]^

While prior investigations on in-hospital cardiac arrests consistently showed temporal variability, with worse outcomes in cases occurring at night,^[4–7]^ the relationship is less consistently shown for OHCA. While several multi-agency studies in Japan^[8,9]^ and United States^[10]^ found poorer outcomes in OHCA occurring at night, the Resuscitation Outcome Consortium (ROC) found that the difference in survival at night disappeared when adjusted for confounders.^[11]^

A further question raised by these studies is whether this apparent temporal variability in OHCA outcomes, if at all present, is a result of mainly disease and physiological factors, modifiable resuscitative efforts, or a combination of both. Some of the key resuscitative elements such as bystander cardiopulmonary resuscitation (CPR) have been found to be less prevalent at night,^[8]^ but these relationships are largely uninvestigated and lacks clarity. Clarity of these trends would identify opportunities for improvements, and would have important implications on resource allocation, in areas of community and public education, EMS staffing and ambulance deployment as well as hospital interventions.

This study aimed to elucidate any differences in OHCA survival during the night compared with during the day, and the factors associated with any differences in survival. We hypothesized that 30-day survival is lower for OHCA occurring at night. We further hypothesize that some modifiable resuscitative characteristics, such as bystander CPR, automated external defibrillator (AED) availability and usage, EMS response time, and provision of post-resuscitation care like emergency percutaneous coronary intervention (PCI), are less optimally delivered at night.

## 2 Materials and methods

### 2.1 Study design—the Pan-Asian Resuscitation Outcomes Study

The Pan-Asian Resuscitation Outcomes Study (PAROS) was established as a multi-center registry to provide baseline information on OHCA epidemiology, management and outcomes, describe variations among EMS systems, and compare systemic and structural interventions in the Asia Pacific.^[12]^

This study used data from twelve sites in seven countries—Singapore, Japan, South Korea, Malaysia, Thailand, Taiwan, and United Arab Emirates. Table 1 shows the characteristics of the twelve participating sites, which made up a total of 808 receiving hospitals and 113 EMS agencies. There were demographic, socioeconomic, and EMS system variations between sites.^[2,13]^ The registry included OHCA of all etiology brought in by EMS or presenting at emergency departments (EDs), as confirmed by the absence of pulse, unresponsiveness, and apnea.

**Table 1:**
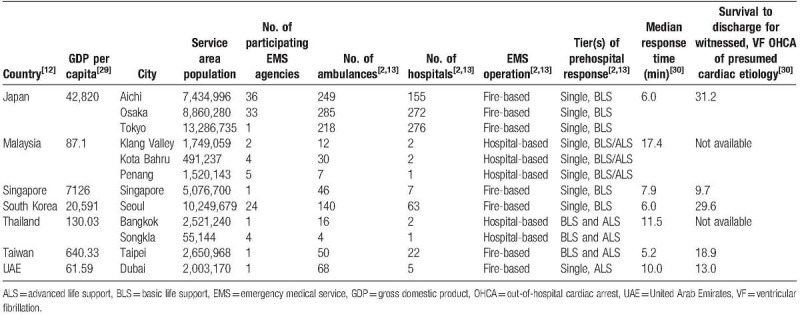
Characteristics of the twelve study sites.

Data definitions follow the Utstein recommendations^[14]^ and collaboration with the Cardiac Arrest Registry to Enhance Survival (CARES)^[15]^ in the United States enabled the development of a unified taxonomy and data dictionary to allow valid comparisons with global data.^[12]^

Each participating country was responsible for administering its own data collection process. Data was extracted from emergency dispatch records, ambulance patient case notes, and ED and in-hospital records. All data were collected with standardized case record forms and data dictionary, and were input via a secured shared internet-based electronic data capture system hosted by the Study Coordination Centre (SCC) in Singapore or via an export field entry process, which auto-populated the PAROS registry.^[12]^

Quality assurance data checks were built into the data entry system, and data verification checks were implemented both at the local sites and the SCC level, to ensure data integrity.^[12]^

### 2.2 Study population

We included all OHCA cases conveyed by EMS or presenting at emergency departments between 2009 and 2012, as confirmed by the absence of pulse, unresponsiveness, and apnea. We excluded cases <18 years of age, those with missing data on time of call or 30-day survival, those of traumatic etiology, and those who had no EMS attempts to resuscitate or resuscitation terminated before arrival at ED. Remaining cases qualified for the main analysis.

### 2.3 Variables and definition

Data definitions follow the Utstein style.^[14]^ Time of OHCA was taken to be the time the call is received at the EMS dispatch center, as this is the first reliable and unambiguous timing from the true time of incident. All cases were then stratified into^[1]^ day (0700H to 1900H) and^[2]^ night (1901H to 0659H the next day). This was derived by dividing the 24 hours in a day into two equal blocks of twelve hours starting from 7:00 AM, where “day” encompasses and corresponds approximately to office hours of most institutions. Time cut-offs based on EMS or hospital shift timings were not feasible due to substantial variations between and within sites.

Response time refers to the interval between time call received by the dispatch center and time of arrival at scene of either the ambulance, or a rapid responder dispatched via the same dispatch center. Scene time refers to the interval between time of arrival at scene and time of leaving the scene. En-route time refers to the interval between time of leaving the location and time of arrival at hospital.

The primary outcome was 30-day survival. The secondary outcomes were other outcome measures such as neurologically favorable survival (defined as Cerebral Performance Category 1 or 2), and modifiable resuscitative characteristics, namely, bystander CPR, bystander AED application, response time, prehospital adrenaline administration, prehospital advanced airway management, emergency PCI or coronary artery bypass graft (CABG), and therapeutic hypothermia.

### 2.4 Statistical analysis

Analyses were performed using R 3.2.2.^[16]^ Patient demographics and OHCA characteristics were summarized and compared by time of call received. Mean and standard deviation were presented for age and time durations and the Mann–Whitney *U* test was used to test the mean differences across time of call received. For categorical variables, frequencies and percentages were described and Chi-square test was used to compare between day and night. Cases with missing data for a particular variable were excluded from analysis.

The effect of night call receipt on the primary outcome of 30-day survival was determined using a logistical regression model, with covariates which were selected based on known predictors of survival from literature and further refined based on results from univariate analysis. Next, the association of night call receipt with differences in modifiable resuscitation characteristics was studied by logistical regression models, each with the delivery of a resuscitative effort being the outcome, namely bystander CPR, bystander AED application, short response time (<8 min), prehospital advanced airway management, prehospital adrenaline, emergency PCI or CABG, and therapeutic hypothermia. The adjusted odds ratio (aOR) and 95% confidence interval (95% CI) of occurrence at night for each resuscitative effort was summarized in a single table. Covariates for each model were selected a priori based on literature and logical reasoning.

### 2.5 Ethics approval

The study was reviewed and approved by local institutional review boards of each participating site.

## 3 Results

### 3.1 Characteristics of study population

**Figure 1:**
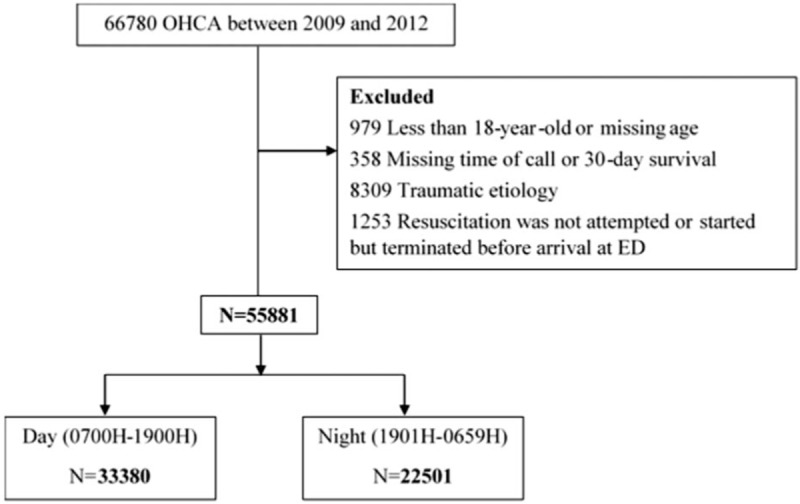
Population flow diagram.

There were 66,780 OHCA cases occurring at the twelve sites between 2009 and 2012. After excluding ineligible cases, 55,881 cases qualified for analysis (Fig. 1).

About 22,501 out of 55,881 cases (40.3%) occurred at night. Mean age was 72.8 years. Overall 30-day survival was 5.7%. Demographics, cardiac arrest characteristics, resuscitation response, and outcomes are shown in Table 2.

**Table 2:**
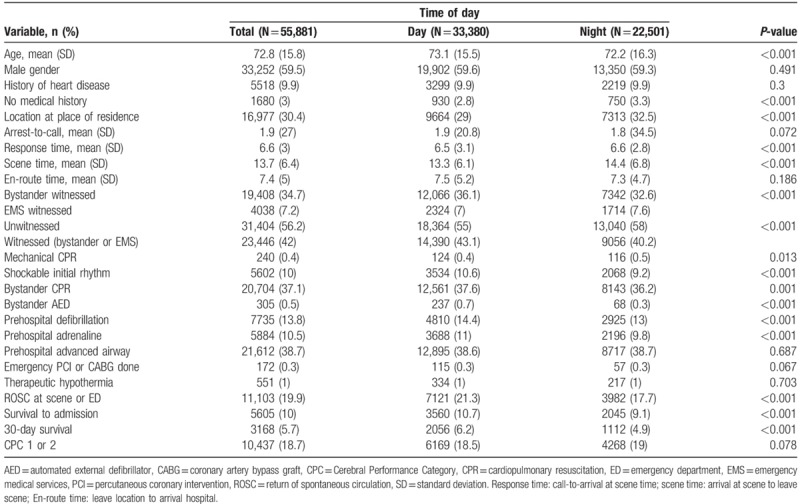
Baseline characteristics, resuscitative characteristics, and outcomes by time of day call received.

In terms of demographics, compared to cases occurring in the day, night cases were younger (72.2 vs. 73.1 years, *P* < .001) and more likely to have no medical history (3.3% vs. 2.8%, *P* < .001),

In terms of cardiac arrest characteristics, compared to cases occurring in the day, night cases were less likely to be witnessed (40.2% vs. 43.1%, *P* < .001), more likely to occur at home (32.5% vs. 29%, *P* < .001), and more likely to have non-shockable initial rhythm (90.8% vs. 89.4%, *P* < .001).

In terms of resuscitation response, compared to cases occurring in the day, night cases had longer mean response time (6.6 vs. 6.5 min, *P* < .001), longer mean on-scene time (14.4 vs. 13.3 min, *P* < .001), more likely to receive mechanical CPR (0.5% vs. 0.4%, *P* < .013), lower bystander CPR rate (36.2% vs. 37.6%, *P* = .001), lower bystander AED application rate (0.3% vs. 0.7%, *P* < .001), lower rate of prehospital defibrillation (13% vs. 14.4%, *P* < .001), and were less likely to receive prehospital adrenaline (9.8% vs. 11%, *P* < .001).

In terms of outcomes, compared to cases occurring in the day, night cases had lower rates of return of spontaneous circulation (ROSC) at ED or at scene (17.7% vs. 21.3%, *P* < .001), lower rates of survival to admission (9.1% vs. 10.7%, *P* < .001), and lower rates of 30-day survival (4.9% vs. 6.2%, *P* < .001).

### 3.2 Incidence of out-of-hospital cardiac arrest by time of call receipt

**Figure 2:**
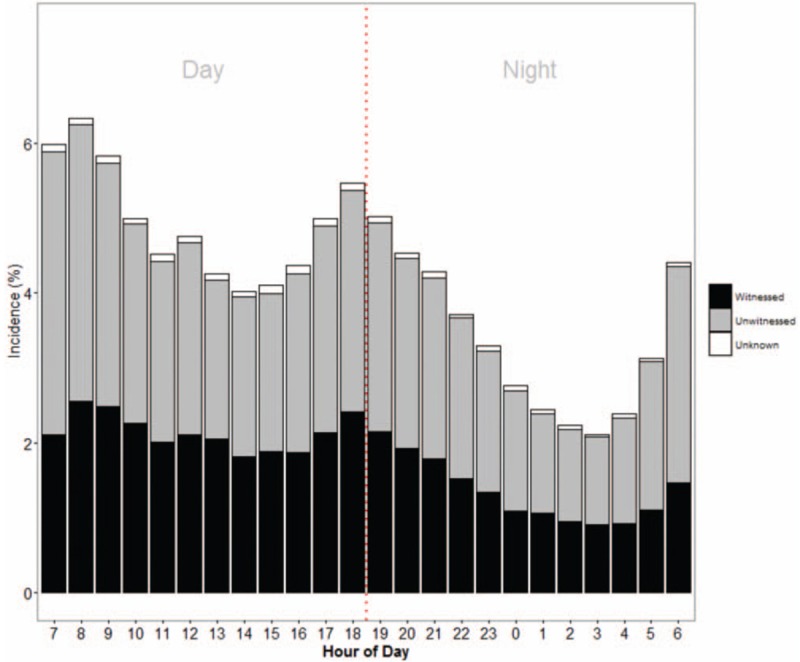
Incidence of out-of-hospital cardiac arrest cases by time of call receipt by dispatch center separated by witnessed and unwitnessed cases.

Figure 2 shows the temporal trend of incidence of out-of-hospital cardiac arrest cases by time of call receipt by dispatch center, for overall, as well as separated by witnessed and unwitnessed cases. Incidence is lower at night (*P* < .001), with a trough at 0300H. There was a large increase from 0700H to 0900H, and a smaller increase from 1700H to 1900H. Both witnessed and unwitnessed cases exhibited this same pattern.

### 3.3 Multivariate analysis of 30-day survival

After adjusting for potential confounders, the probability of 30-day survival for OHCA occurring at night was significantly lower than in day time after controlling for the influence of other predictor variables, with an aOR of 0.79 (95% CI 0.73–0.86, *P* < .001) (Table 3). From the multiple logistic model fitting result, the other variables having a negative effect on 30-day survival were older age, female gender, prehospital adrenaline administration and longer response time, and those having a positive effect were bystander CPR, prehospital defibrillation, shockable initial rhythm, witnessed arrest, emergency PCI, or CABG and therapeutic hypothermia.

**Table 3:**
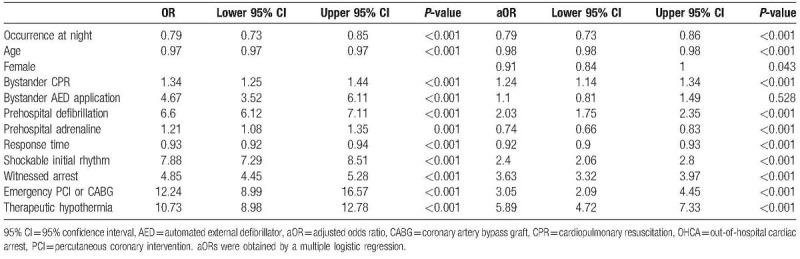
Adjusted odds ratio and 95% confidence intervals of OHCA occurring at night compared to day time for 30-day survival.

### 3.4 Distribution of 30-day survival of out-of-hospital cardiac arrest cases by time of call receipt

**Figure 3:**
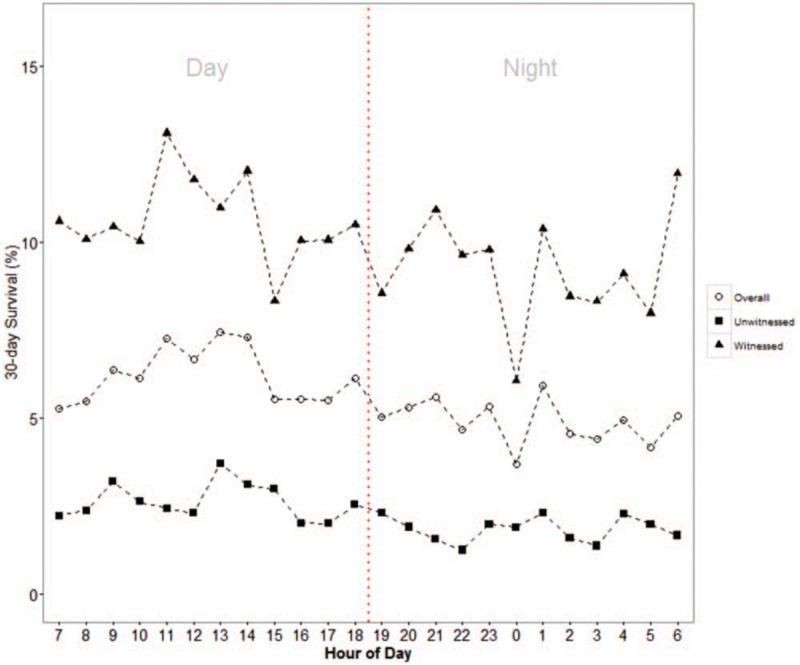
Trend of 30-day survival of out-of-hospital cardiac arrest cases by time of call receipt by dispatch center, for overall, as well as separated by witnessed and unwitnessed cases.

Figure 3 shows the overall variation of 30-day survival of OHCA cases by time of call receipt by dispatch center separated by witnessed and non-witnessed cases. Lower 30-day survival is observed for night cases, with a large trough at midnight. There is a large peak in 30-day survival at 1300H and another at 1100H. These peaks are much more pronounced when considering only witnessed cases and diminishes when considering only unwitnessed cases. The trough at midnight disappears in the unwitnessed group.

### 3.5 Multivariate analysis of modifiable resuscitative characteristics

From univariate logistical regressions, occurrence at night was associated with decreased provision of bystander CPR, bystander AED application, and prehospital adrenaline (Table 4).

**Table 4:**
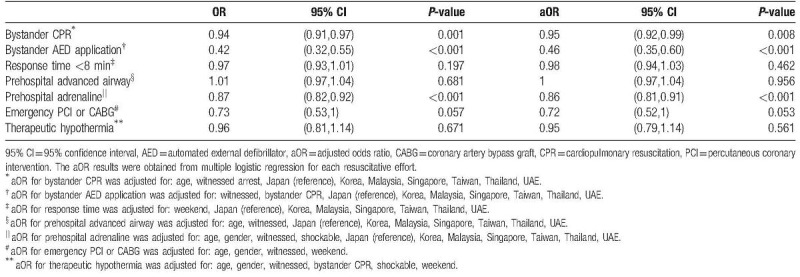
Odds ratio (OR) of OHCA occurring at night compared to day time for modifiable resuscitative efforts.

After controlling for the influence of other factors (variables are listed in the footnote of Table 4) in seven separate logistical regression models, each with a modifiable resuscitative effort as the outcome and night occurrence as a variable, the same three resuscitative efforts were significantly decreased for occurrence at night. Night occurrence was predictive of decreased bystander CPR with aOR of 0.95 (95% CI 0.92–0.99, *P* = .008), decreased bystander AED application with aOR of 0.46 (95% CI 0.35–0.60, *P* < .001), and decreased prehospital adrenaline administration with aOR of 0.86 (95% CI 0.81–0.91, *P* < .001).

## 4 Discussion

### 4.1 Circadian variation in incidence

In this cohort of Pan-Asian OHCA cases treated by developing or newly developed EMS, several findings emerged. First, we observed a circadian pattern similar to that found in existing literature,^[10]^ with a large increase from 0700H to 0900H, and a smaller increase from 1700H to 1900H. However, we did not find a difference in trends when comparing the witnessed and unwitnessed group separately, which disputes the postulation by Bagai et al that these peaks may have been attributable to cases that died in the night or during working hours and were only discovered when family members awaken or return home from work respectively.^[10]^ If this were to be the case, then the peaks should greatly diminish when considering only the witnessed group. Indeed, extrapolating from studies on myocardial infarction on chronobiological phenomenon such as heart rate, vascular tone, heart rate variability or hormonal changes,^[17,18]^ physiological reasons may be a primary reason for the circadian variation found in our study.

### 4.2 Circadian variation in 30-day mortality

30-day mortality was higher in OHCA cases occurring at night even after adjusting for confounders. This affirms some prior studies that found poorer outcomes for cases occurring outside office hours and particularly overnight.^[9–11,19,20]^ We further found the increased mortality at night, along with its patterns of peaks and troughs, diminished greatly when considering only unwitnessed cases. In fact, the large trough at midnight completely disappears in the unwitnessed group. This is a novel analysis and finding. The witnessed group represents cases that were discovered shortly after the true arrest time, as opposed to the unwitnessed group which has less reliable time of arrest, and even be found dead several hours after the fact. A possible explanation is that since the unwitnessed group had worse prognosis to begin with (due to long downtime), any difference in resuscitative efforts by EMS and hospitals had reduced effect sizes. Hence these cases did not exhibit the expected circadian pattern.

### 4.3 Resuscitative efforts at night

We found that occurrence at night was independently associated with decreased provision of bystander CPR, bystander AED application, and prehospital adrenaline, after adjusting for confounders in separate multivariate regressions. This shows some concordance with Matsumura et al,^[8]^ who reported a multi-agency study of 16,164 OHCA cases in the Kanto region of Japan and found lower bystander CPR at night, but no difference in adrenaline administration and defibrillation.

The weakest link here appears to be bystander efforts. This is an interesting observation, as it represents an opportunity for improvement in increasing the provision of these important treatment modalities at night. This is crucial as bystander CPR and AED application confer large survival benefits.^[21,22]^ Education of the public, and especially the family members of at risk patients, may increase vigilance for cardiac events that occur overnight. AED availability at night is expected to be lower as public access defibrillator programs generally find better cost effectiveness in placing AEDs outside residential premises.^[23]^ Probability of an OHCA patient receiving bystander CPR is also lower because he does not have access to a pool of passers-by, some of whom may be trained and willing to provide CPR. These are strong impetuses for the provision of training targeted at the household of at-risk patients. In addition, mobile phone applications, such as the myResponder app used in Singapore, has potential value in improving bystander CPR and AED application by activating community resources, such as alerting the user to the location of the nearest AED, and by broadcasting the cardiac arrest case to a network of volunteers trained in CPR/AED who are in the vicinity. The development of national AED registries may serve to elucidate geographic coverage of AEDs with relation to OHCA incident locations and this may inform policy-making with regards to AED deployment.

While adrenaline has not been well linked to improvements in patient-oriented outcomes,^[24]^ it is recommended in contemporary resuscitation guidelines.^[25]^ The failure to administer adrenaline may suggest that unmeasured resuscitative efforts, might also have been less well delivered (perhaps qualitatively). An example of unmeasured resuscitative characteristics would be quality of CPR performed. Also, the provision of key resuscitative measures is largely a quality assurance matter and may well vary between EMS agencies. As such, it is possible that significant differences were cancelled out in analyses of pooled data from multiple agencies. It has been shown that wide variations in OHCA survival outcomes exist between communities, and it has been strongly suggested that these differences are mainly due to how pre-hospital emergency care is delivered.^[26]^ This finding, coupled with that of longer EMS response time at night, provides an impetus for EMS systems to review quality assurance for consistency of care at night.

That the modifiable resuscitative efforts, which are known to confer survival benefit, are poorer at night, would at least partially explain the reduced survival at night. It is however not known whether there are also disease-specific factors and physiological factors that play important roles leading to poorer outcomes at night. While the literature on the physiological differences in night OHCAs is limited, studies on myocardial infarction may lend generalizable principles. Chronobiological phenomenon such as changes in hemodynamics, hormones, coronary blood flow, and platelet aggregation may be implicated.^[18]^ While this study suggests that disease and physiological factors play a prominent role, the relative burden of these modifiable efforts and disease factors remain unknown, and human studies investigating this would necessarily have to examine severity markers of physiological, serological, and angiographic sources.

### 4.4 Strengths and limitations of this study

The strength of this study lies in its multinational multi-agency design which allows the pooling of standardized data from a variety of cultural backgrounds, EMS, and hospital systems, across seven countries. This allows a breadth of systems and a mix of both developing and newly developed EMS system. The resultant large number of EMS systems represented allows the randomization of unaccounted factors that are system-related, such as shift timings and shift staffing adequacy.

This study suffers from several limitations. First, there may be unmeasured qualitative modifiable resuscitation characteristics that mediated the poorer outcomes for night OHCA. Shift work disrupts circadian rhythms and may influence resuscitation performance when attention and concentration is hindered.^[27,28]^ Night shifts may also be less well staffed in some EMS systems, similarly resulting in performance differences.^[29,30]^ The data captured by many cardiac arrest registries lack the granularity to provide information on factors related to performance, such as CPR quality.

Secondly, this study did not examine in detail in-hospital resuscitative characteristics. While factors that are known to have sizeable effect sizes on outcomes (PCI, therapeutic hypothermia) were included in the multivariate model, clearly other efforts exert prognostic implications. For example, Matsumura et al discovered lower odds of in-hospital intubation and blood-gas analysis for night OHCA.^[8]^ The relatively low rates of PCI and therapeutic hypothermia in this cohort may not have powered a demonstration of the difference at night.

Thirdly, the observational nature of this study precludes the conclusion of causal links between the found associations of poorer resuscitative efforts and poorer outcomes.

Fourthly, the use of call receipt time as a surrogate for time of occurrence is not without disadvantages, as there is a delay between the true time of occurrence and the actual call. This delay may be larger in cases at night owing to delay in discovery by bystanders. However, we elected not to use onset time due to expected recall bias. We performed subgroup analyses for the witnessed cases as these cases are subjected to less discordance between time of call receipt and true arrest time.

## 5 Conclusion

In this international cohort, 30-day survival was worse in OHCA occurring at night. There were circadian patterns in incidence. Bystander CPR and bystander AED application were significantly lower at night in the multivariate analysis. This would at least partially explain the decreased survival at night.

## 6 PAROS clinical research network

Participating Site Investigators: AK Sarah and MN Julina (Hospital Sungai Buloh, Selangor, Malaysia), NAR Hisamuddin (School of Medical Sciences, Universiti Sains Malaysia, Kelantan, Malaysia), GY Naroo (Rashid Hospital, Dubai, United Arab Emirates), AS Omer and T Yagdir (Dubai Corporation for Ambulance Services, Dubai, United Arab Emirates), N Khunkhlai (Rajavithi Hospital, Bangkok, Thailand), A Monsomboon (Siriraj Hospital, Bangkok, Thailand), T Piyasuwankul (Prince of Songkla University, Hatyai, Thailand), T Nishiuchi (Kindai University Faculty of Medicine, Osaka, Japan), K Kajino (Critical Care Medical Center, Osaka National Hospital, Osaka, Japan), T Nakagawa (Aichi Medical University Hospital, Aichi, Japan), PCI Ko (National Taiwan University Hospital, National Taiwan University, Taipei, Taiwan), Hyun Wook Ryoo (School of Medicine, Kyungpook National University, Daegu, Korea), KJ Song (College of Medicine, Seoul National University, Seoul, Korea), DRH Mao and ES Goh (Khoo Teck Puat Hospital, Singapore), LP Tham (KK Women's & Children's Hospital, Singapore), SO Cheah (Ng Teng Fong General Hospital, Singapore), MYC Chia (Tan Tock Seng Hospital, Singapore), HN Gan and L Tiah (Changi General Hospital, Singapore), BSH Leong (National University Hospital, Singapore).

## Acknowledgments

We acknowledge the contributions of Singapore Clinical Research Institute for secretariat support, study coordination, and data management.

## Author contributions

**Conceptualization:** Andrew Fu Wah Ho, Marcus Eng Hock Ong.

**Data curation:** Andrew Fu Wah Ho.

**Formal analysis:** Andrew Fu Wah Ho, Ying Hao.

**Investigation:** Andrew Fu Wah Ho.

**Methodology:** Andrew Fu Wah Ho.

**Supervision:** Marcus Eng Hock Ong.

**Validation:** Ying Hao, Marcus Eng Hock Ong.

**Writing – original draft:** Andrew Fu Wah Ho, Ying Hao, Pin Pin Pek, Nur Shahidah, Susan Yap, Yih Yng Ng, Kwanhathai Darin Wong, Eui Jung Lee, Pairoj Khruekarnchana, Win Wah, Nan Liu, Hideharu Tanaka, Sang Do Shin, Matthew Huei-Ming Ma, Marcus Eng Hock Ong.

**Writing – review & editing:** Andrew Fu Wah Ho, Ying Hao, Pin Pin Pek, Nur Shahidah, Susan Yap, Yih Yng Ng, Kwanhathai Darin Wong, Eui Jung Lee, Pairoj Khruekarnchana, Win Wah, Nan Liu, Hideharu Tanaka, Sang Do Shin, Matthew Huei-Ming Ma, Marcus Eng Hock Ong.
